# Optimal ranges of variables for an effective adsorption of lead(II) by the agricultural waste pomelo (*Citrus grandis*) peels using Doehlert designs

**DOI:** 10.1038/s41598-018-19227-y

**Published:** 2018-01-15

**Authors:** Xiao-Lan Yu, Yong He

**Affiliations:** College of Biosystems Engineering and Food Science, Zhejiang University, Hangzhou, 310058 P. R. China

## Abstract

The capacity of pomelo peels’ adsorption on lead(II) from aqueous solutions without modifications was investigated and confirmed. Four variables in this study, pH, temperature, time and initial concentration of lead(II), significantly affected the adsorption rate of pomelo peels. The prediction model and optimal ranges of optimized variables were given by Doehlert designs, which made the selection of variables rapid, flexible and effortless to obtain an adsorption rate reaching 99.9% and 20 mg/L for initial lead(II) concentration, 3 for pH, 50 °C for temperature and 210 min for time was a choice. The higher correlation coefficient as well as the more consistent value of experimental equilibrium adsorption capacity of the pseudo-first-order model suggested it bore a better prediction of the adsorption kinetics than the pseudo-second-order model. Langmuir model indicated the adsorption mechanism of pomelo peels was monolayer sorption with the help of both physical adsorption and chemical bonding, which were demonstrated by scanning electron microscopy and Fourier transform-infrared, respectively. The ability of pomelo peels to adsorb lead(II) from aqueous solutions was not interfered with the presence of calcium(II), magnesium(II), copper(II) and zinc(II). Pomelo peels had the potential to be utilized in the simultaneous adsorption of toxic heavy metal ions.

## Introduction

Removing toxic heavy metal ions from aqueous solutions is still a key issue in the management of environmental pollution. Mercury (Hg), cadmium (Cd), chromium (Cr), arsenic (As), lead (Pb) and nickel (Ni) are major contributors to poisonousness in wastewater, while among these ions, lead(II) is more likely to cause people’s attention because of its cumulative toxicity, extensively existence and various sources^1–3^. According to World Health Organization (WHO), lead exposure is estimated to account for 0.6% of the global burden of disease, with the highest burden in developing regions^4^, which makes the removal of lead(II) from wastewater of great urgency and importance.

Conventional methods, such as chemical precipitation, ion exchangers, chemical oxidation/reduction, reverse osmosis, electro dialysis and ultrafiltration^5^, with widely acceptance and application, have their inherent limitations of less efficiency, sensitive operating conditions and costly instruments^6^, however, adsorption, especially adsorption by agricultural wastes as bio sorbents go pass these limitations^7–15^. Using agricultural wastes as bio sorbents on heavy metal ions removal not only benefits sewage treatments, but also contributes to the recycle of agricultural wastes. Pomelo (*Citrus grandis*), a natural (non-hybrid) citrus fruit with a much thicker rind, is similar in appearance to a large grapefruit and widely cultivated in eastern and south-eastern China. The peel of the pomelo accounts for more than 50% of the total weights, tastes bitter, considered inedible, and thus is usually discarded. Notwithstanding, it’s appropriate to describe pomelo peel as a new, low cost, abundantly available adsorbent due to its application in the adsorption of dyes^16–19^ and oil pollution^20,21^ with or without modifications or carbonizations. Satisfactory effects in these researches present the potential ability of pomelo peels to be utilized in the adsorption of lead(II) in wastewater, which is less information available at present^22,23^ and all researchers used modified pomelo peels instead of non-modified pomelo peels. Modifications and carbonizations can indeed improve the adsorption ability of agricultural wastes, whereas, they add extra procedures of the adsorption processes plus time and chemical reagents. Once the cost of pretreatment weighs more than the increase of adsorption ability, the most advantage of agricultural wastes to be applied as bio sorbents is lost.

According to current studies, the adsorption rate of bio sorbents is affected by a variety of factors, for example, the pH of the solution, temperature and time of the adsorption, the amount of bio sorbents and initial concentration of toxic heavy metal ions. In comparison with Doehlert designs^24^, one of response surface methods which are more efficient and offer advantages in relation to central composite^13,14^ and Box–Behnken designs^25^, one-variable-at-a-time, the method that mostly applied for the optimization of factors^11,12,18,19^ in bio sorbents’ adsorptions loses sight of the potential interactions between experimental variables as well as needs more time and experiments to optimize the whole factors. Besides, different from one-variable-at-a-time, Doehlert designs give the prediction model of the adsorption rate, through which the adjustment of some variables with others remaining the same is accessible and makes the optimized variables could be in a range rather than a single point, facilitating the industrial use of agricultural wastes as bio sorbents.

To investigate the capacity of pomelo peels’ adsorption on lead(II) removal without modification or carbonization, to explore the potential interactions between four experimental variables, which were pH, temperature, time and initial concentration of lead(II), and to provide quantitative effects, optimal ranges as well as the prediction model at the same time, Doehlert designs were selected and conducted in this study. Adsorption kinetic and isotherm models were fitted to discuss the mechanism of pomelo peel’s adsorption. Then, interfering study was carried out to test the selectivity of pomelo peels. The dominant functional groups were discovered by Fourier transform-infrared (FT-IR).

## Results and Discussion

### Adsorption performances

#### Significant variables

Analytical results demonstrated that the linear model of lead(II) removal by pomelo peels as well as the whole four variables were significant, seen in Table 1, without significant interactions.

**Table 1:** Analysis of variance (ANOVA) for the linear model of lead(II) removal by pomelo peels.

Source	Sum of Squares	d_*f*_^1^	Mean Square	*F*-value	Probability >F
Model	9485.60	4	2371.40	13.20	<0.0001
*A: pH*	2390.17	1	2390.17	13.31	0.0018
*B: temperature*	1575.53	1	1575.53	8.77	0.0083
*C: time*	4453.38	1	4453.38	24.80	<0.0001
*D: Pb(II) concentration*	1463.81	1	1463.81	8.15	0.0105
Residual	3232.72	18	179.60		
*Lack of Fit*	3232.35	16	202.02	1084.78	0.0009
*Pure Error*	0.37	2	0.19		

^1^d_*f*_ = degree of freedom.

**Figure 1:**
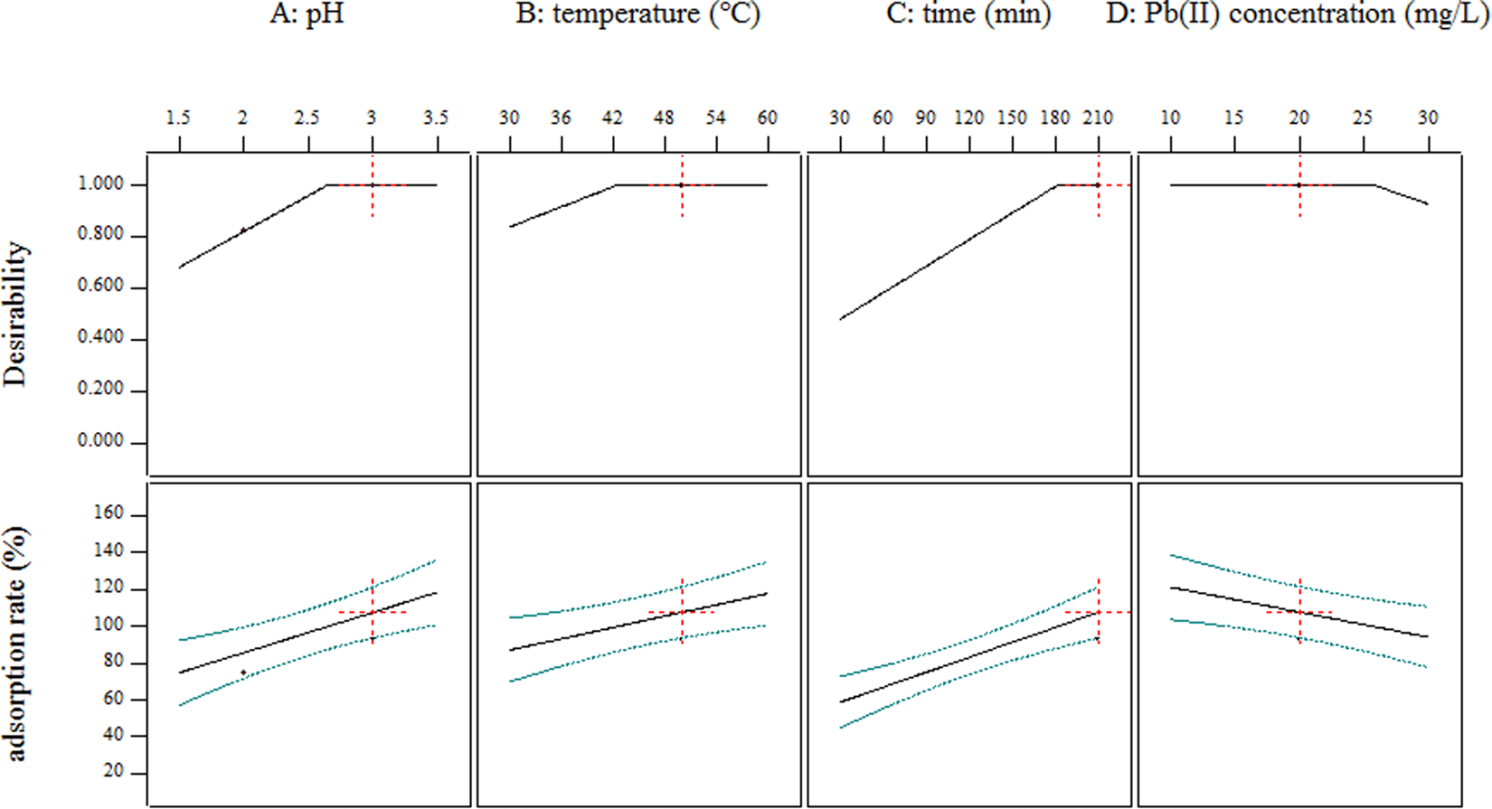
Quantitative effects and optimal ranges of pH, temperature, time and initial lead(II) concentration for lead(II) removal by pomelo peels.

Quantitative effects and optimal ranges of these four significant variables were illustrated in Fig. 1. Dotted red crosses in Fig. 1 provided a choice for the adsorption rate approximately reaching 100%, that was, 20 mg/L for initial lead(II) concentration, 3 for pH, 50 °C for temperature and 210 min for time, respectively.

It was obvious that in the range of this study, the adsorption rate of lead(II) on pomelo peels increased as the pH, temperature and time increased, while as the initial lead(II) concentration decreased. Trends of temperature, time and initial lead(II) concentration were consistent with existing researches^7,8,12,26–28^; the adsorption process through pomelo peels required time to arrive at equilibrium and a higher temperature allowed the process to reach equilibrium more rapidly; also, the binding sites in pomelo peels for lead(II) were limited so that when the initial lead(II) concentration was at a certain value, pomelo peels were going to reach saturation and would no longer absorb lead(II) from aqueous solutions.

As for pH, situation was not similar. Due to the upper limit of pH in this study was 3.5, the optimum of pH for lead(II) removal with the value of 5.0 or 6.0 proposed by other researches^29^ did not occur; neither did the tendency with an ascent at first followed with a decline afterwards. Nevertheless, based on the trend of pH in Fig. 1, it was appropriate to infer that the optimal value of 5.0 or 6.0 in other researches was in the optimal ranges of pH in this study. The upper limit of pH with the value of 3.5 was selected for the following two reasons: i) to investigate the feasibility of a lower pH for lead(II) removal through pomelo peels; ii) to make the optimal pH for lead(II) removal closer to other heavy metal ions’ optimums, which was benefit to the simultaneous adsorption.

#### Prediction model

The prediction model given by Doehlert designs was presented as the following:

$${\rm{adsorption}}\,{\rm{rate}}=-38.78+21.86\,\times {\rm{pH}}+1.02\times {\rm{temperature}}+0.27\times \text{time}\,-1.36\,\times {\rm{Pb}}({\rm{II}}){\rm{concentration}}$$

It was of convenience to choose the corresponding values or ranges for other three variables accurately based on the distinct initial lead(II) concentrations to acquire a higher adsorption rate by using the linear model. Moreover, the steady segments in *Desirability* of pH, temperature and time along with the segment of adsorption rate above 100% in Fig. 1 showed that to achieve the purpose of adsorption rate close to 100% in various initial lead(II) concentrations ranging from 10 mg/L to 25 mg/L, it was feasible to adjust two variables like pH and temperature with the other one like time remain the same, which made the adsorption process flexible and controllable in practical applications. Also, it was helpful to apply pomelo peels in the simultaneous adsorption of more than two toxic heavy metal ions since the optimum of pH was usually changed as metal ions changed, like 2.0 for chromium(VI)^30^, 4.0 for arsenic(V)^31^ and 6.0 for cadmium(II)^27^.

### Adsorption kinetics

**Figure 2:**
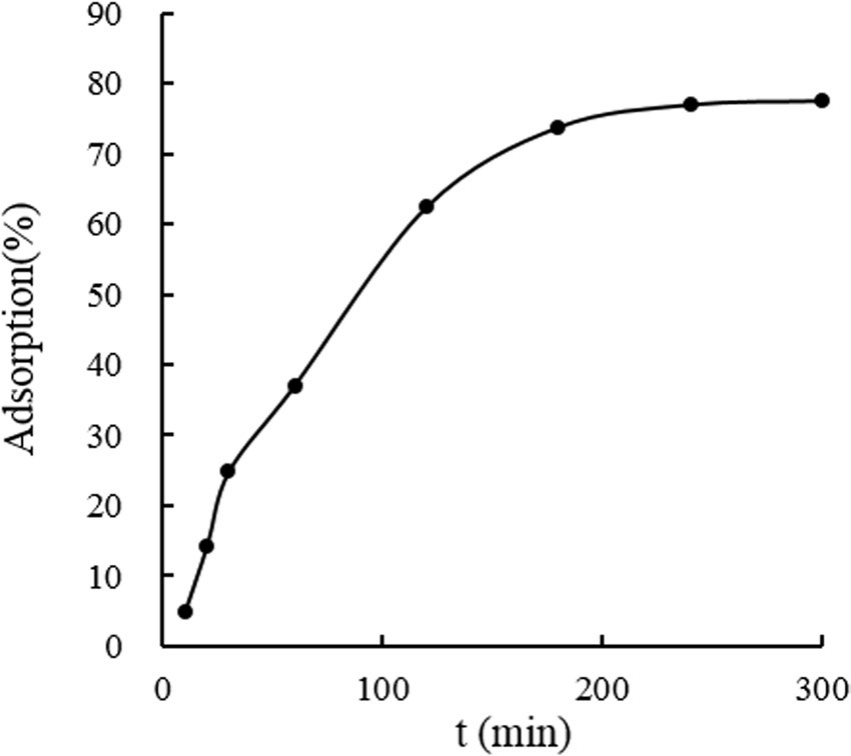
Adsorptions kinetics for lead(II) removal by pomelo peels at 45 °C with pH of 3.

Figure 2 demonstrated the kinetics of the adsorption of 20 mg/L lead(II) by pomelo peels at 45 °C with pH of 3. Sharply increased at the initial stage (0–30 min) of adsorption, the amount of lead(II) adsorpted by pomelo peels gradually increased at the middle stage (30–120 min) and reached an equilibrium value in approximately 210 min. Thus, the adsorption time was fixed at 210 min for the rest experiments to assure the equilibrium was arrived at.

From Table 2, parameters presented that the pseudo-first-order model was followed better than the pseudo-second-order model not only the *R*^2^, but also the consistence between the *q*_e_ (cal.) and *q*_e_ (exp.), which was in accordance with the existed research^8^ using the peel of *Citrus reticulata* without modification as the bio sorbent.

**Table 2:** Kinetic parameters for the adsorption of lead(II) by pomelo peels at 45 °C with pH of 3.

*q*_e_ (exp.) (mg/g)	Pseudo-first-order			Pseudo-second-order		
	*q*_*e*_ (cal.) (mg/g)	*k*_1_ (min^−1^)	*R* ^2^	*q*_*e*_ (cal.) (mg/g)	*k*_2_ (g mg^−1^ min^−1^)	*R* ^2^
1.549	1.719	0.0122	0.9946	3.0609	0.00159	0.7785

### Adsorption isotherms

**Figure 3:**
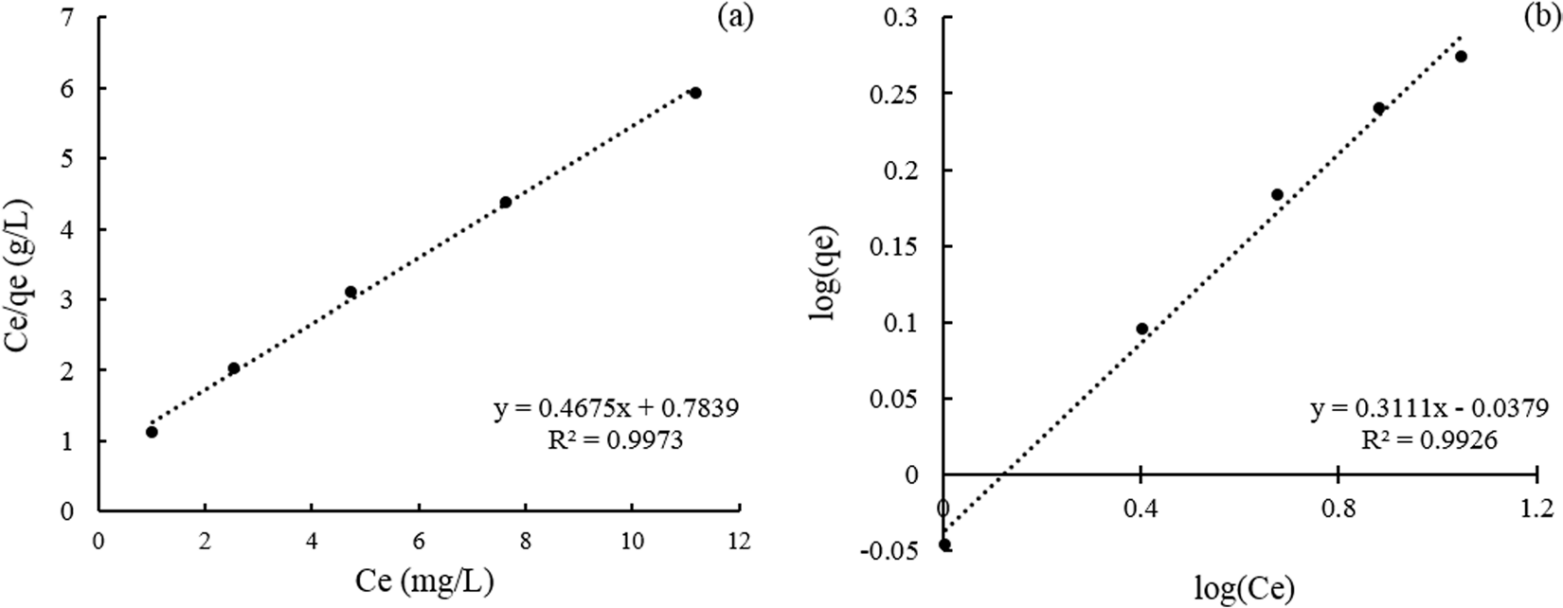
Isotherm plots for lead(II) removal by pomelo peel at 30 °C with pH of 2.5. (**a**) Langmuir model; (**b**) Freundlich model.

In isotherm models, Langmuir model fitted better than Freundlich model and model parameters were listed in Table 3 with plots given in Fig. 3. Valid for monolayer sorption onto a surface with a finite number of identical sites, which were homogeneously distributed over the adsorbent surface, Langmuir model indicated the adsorption mechanism of pomelo peels.

**Table 3:** Isotherm parameters for the adsorption of lead(II) by pomelo peels at 30 °C with pH of 2.5.

Langmuir model				Freundlich model	
*q*_*max*_ (mg/g)	*b* (L/mg)	*R* ^2^	*K*_*F*_	*n*	*R* ^2^
2.139	0.5964	0.9973	0.9164	3.2144	0.9926

The value of *R*_*L*_ pointed out the type of Langmuir isotherm to be irreversible (*R*_*L*_ = 0), favourable (0 < *R*_*L*_ < 1), linear (*R*_*L*_ = 1) or unfavourable (*R*_*L*_ > 1). In this study, *R*_*L*_ was found to less than 0.3 for lead(II) concentration of 5 mg/L and less than 0.015 for 100 mg/L, which presented that pomelo peel was an efficient adsorbent with satisfactory adsorption effect in the removal of lead(II) from aqueous solutions, even without modifications or carbonizations.

### Interfering study

Compared the result of interfering study, absorption rate (int.), with adsorption rate (exp.) obtained from the kinetic experiment which shared the same experiment condition, seen in Table 4, it was concluded that the presence of calcium(II) and magnesium(II) at 100 times concentration and the presence of copper(II) and zinc(II) at the same concentration had no interference with the adsorption of lead(II) by pomelo peels.

**Table 4:** Results of interfering study.

Concentration (mg/L)		Adsorption rate (exp.) (%)	Adsorption rate (int.) (%)	Relative error (%)
Ca(II), Mg(II)	Cu(II), Zn(II), Pb(II)			
200	20	76.90	74.40 ± 0.4822	3.25

### SEM analysis

**Figure 4:**
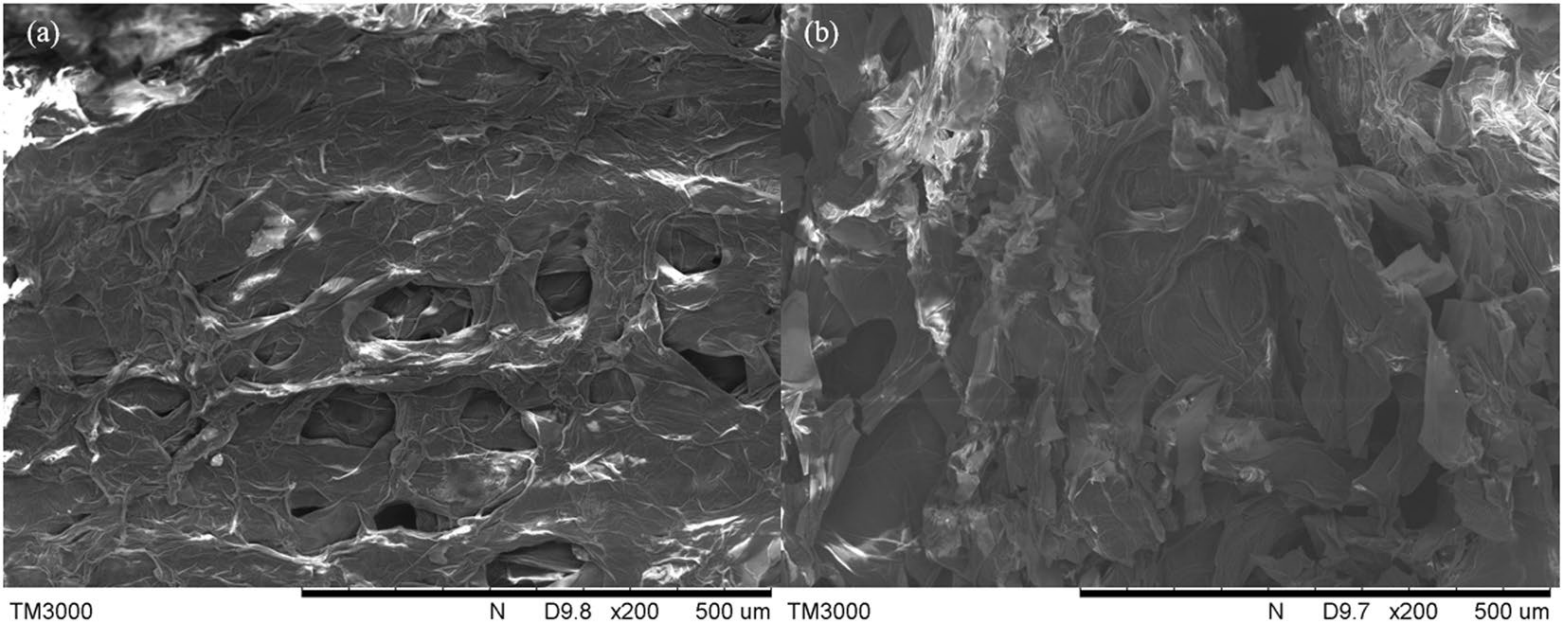
SEM micrographs of pomelo peels. (**a**) Before adsorption; (**b**) After adsorption at 45 °C with pH of 3.

Before adsorption, plenty of even, developed and well pronounced pores were over the surface of pomelo peels, as Fig. 4(a) demonstrated, forming an orderly pore structure. However, after adsorption, the orderly pore structure of pomelo peels was destroyed or disappeared to a certain extent, illustrated in Fig. 4(b). SEM micrographs of pomelo peels before and after lead(II) adsorption suggested that adsorption might be conducted by physical adsorption due to pomelo peels’ pore structure, though, interfering study’s result also indicated the possibility of chemical bonding, which was evaluated and confirmed by FT-IR analysis.

### FT-IR analysis

**Figure 5:**
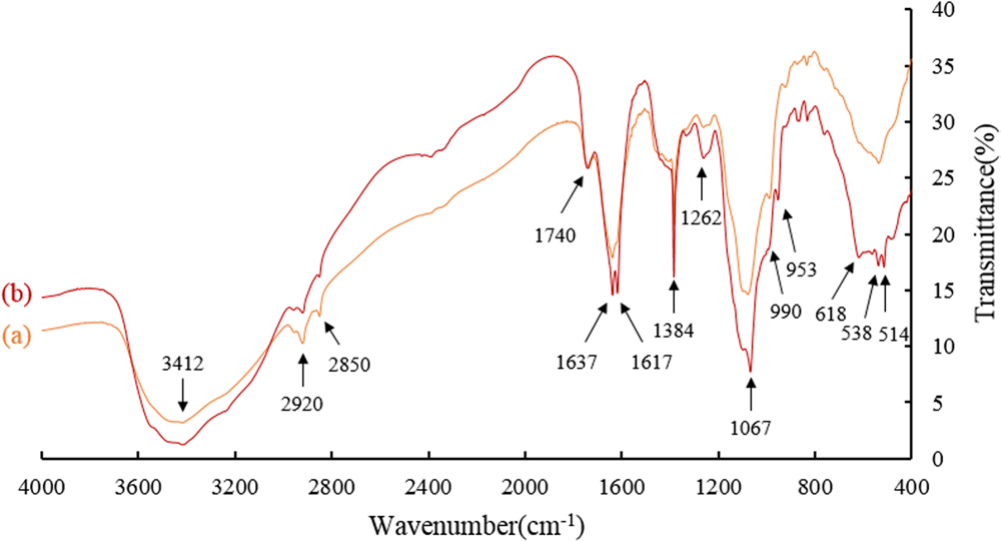
FT-IR spectra of pomelo peels. (**a**) Before adsorption; (**b**) After adsorption at 45 °C with pH of 3.

The pattern of sorption of metals onto plant materials was attributable to the active groups and bonds present on them^32^. FT-IR spectroscopy was, therefore, carried out for preliminary qualitative analysis of major functional groups worked in the adsorption of lead(II) by pomelo peels. From Fig. 5, differences of spectrum (a) and spectrum (b) were classified into two categories: i) changes of peaks intensities and ii) generations of peaks. As Fig. 5 showed, peak 3412 cm^−1^, 2920 cm^−1^, 2850 cm^−1^, 1637 cm^−1^, 1384 cm^−1^, 1262 cm^−1^, 1067 cm^−1^ and 538 cm^−1^ belonged to the first category, while peak 1617 cm^−1^, 990 cm^−1^, 618 cm^−1^ and 514 cm^−1^ were classified to the second category. The broad absorption peak at 3412 cm^−1^ might be correspond to the O–H stretching vibrations of cellulose, pectin and lignin^18,33^. Peaks of 2920 cm^−1^ and 2850 cm^−1^ were assigned to symmetric vibration of CH_n_ especially C–CH_2_ bonds^18^, peak 1637 cm^−1^, 1384 cm^−1^, 1262 cm^−1^, 1067 cm^−1^ and 538 cm^−1^ could be related to ketones, -CH_3_ in alcohols and phenols, secondary amides, -C-O stretching vibrations of primary alcohols and C-Br^34^. New generated peaks 1617 cm^−1^, 990 cm^−1^, 618 cm^−1^ and 514 cm^−1^ might be regarded as intermolecular hydrogen bonds in ketones, alkene mono-substitution and C-Cl according to current literatures^34^. Hydroxyl groups in biopolymers were considered as proton donors, hence, deprotonated hydroxyl groups may be involved in coordination with metal ions^35^, so did in this research.

## Conclusions

To conclude, Doehlert designs were applied for the test of pomelo peels’ adsorption capacity without modifications for lead(II) removal. Pomelo peels, one kind of agricultural wastes, enjoyed the advantages of easy acquisition and simple pre-treatment, and performed outstandingly in lead(II) adsorption, which provided a new way for their reuse. Four variables in this research, pH, temperature, time and initial concentration of lead(II) were significant and the linear model offered by Doehlert designs made the selection of variables effortless and flexible to acquire an adsorption rate more than 99.9% due to the optimal range it provided rather than a single point of optimized variables. Adsorption kinetic studies presented the adsorption process came to an equilibrium at 210 min approximately with a better fitting of the pseudo-first-order model. Isotherm models of Langmuir showed that the mechanism of pomelo peels’ adsorption was monolayer sorption. Taking the results of SEM analysis and FT-IR analysis into account, the adsorption of lead(II) by pomelo peel was the the combination of physical adsorption and chemical bonding. The presence of calcium(II), magnesium(II), copper(II) and zinc(II) in aqueous solutions would not interfere the absorption of lead(II) by pomelo peels. This work demonstrated that the pomelo peel was an effective adsorbent for removing lead(II) from aqueous solutions without modification and Doehlert designs were of great use for variables optimization of bio sorbents’ adsorptions. Experiments on investigating the adsorption ability of pomelo peels to remove other toxic heavy metal ions from aqueous solutions with lead(II) simultaneously were in progress in our laboratory. Easy and low-cost modifications of pomelo peels were considered at the same time.

## Materials and Methods

### Chemicals

All chemicals and reagents used in this research were of analytical grade and bought from Sinopharm Chemical Reagent Co., Ltd, Shanghai, P. R. China. Deionized water (≥18.2 MΩ) was applied for the preparation of aqueous solutions.

Lead(II) solutions with various concentrations were prepared by mixing appropriate amounts of Pb(NO_3_)_2_ and deionized water. 0.1 M HNO_3_ or NaOH was employed to adjust the pH of lead(II) solutions with the help of Sartorius PB-10 pH meter.

### Adsorbent preparations

Pomelos were acquired from Yuhuan County, Zhejiang Province, P. R. China, and their peels were washed with distilled water several times and immersed in deionized water for 24 hours to eliminate the interference effect of other particles. Then pomelo peels were dried in a hot air oven at 60 °C^18^ until they were in constant weights and stored in the desiccator.

The amount of pomelo peels in this study was 0.5 g in the dry state through cutting and weighting by the balance. The volume of lead(II) solutions was 50 mL per experiment.

### Adsorption experiments

#### Doehlert designs

A Doehlert matrix for four-variables^25^ was selected and details were shown in Table 5. Ranges of variables were designed on the basis of current literatures.

**Table 5:** Doehlert designs, experimental plan and adsorption rates (*Y*).

	Doehlert designs					Experimental plan			*Y*
	A	B	C	D	pH	temperature ( °C)	time (min)	initial Pb^2+^ (mg/L)	
1	1	0	0	0	3.5	45	120	20	75.56
2	0.5	0.866	0	0	3	60	120	20	99.90
3	0.5	0.289	0.817	0	3	50	210	20	92.80
4	0.5	0.289	0.204	0.791	3	50	150	30	72.15
5	−1	0	0	0	1.5	45	120	20	47.05
6	−0.5	−0.866	0	0	2	30	120	20	49.80
7	−0.5	−0.289	−0.817	0	2	40	30	20	21.05
8	−0.5	−0.289	−0.204	−0.791	2	40	90	10	24.12
9	0.5	−0.866	0	0	3	30	120	20	62.08
10	0.5	−0.289	−0.817	0	3	40	30	20	51.06
11	0.5	−0.289	−0.204	−0.791	3	40	90	10	99.90
12	−0.5	0.866	0	0	2	60	120	20	84.74
13	0	0.577	−0.817	0	2.5	55	30	20	35.25
14	0	0.577	−0.204	−0.791	2.5	55	90	10	99.00
15	−0.5	0.289	0.817	0	2	50	210	20	74.56
16	0	−0.577	0.817	0	2.5	35	210	20	92.15
17	0	0	0.613	−0.791	2.5	45	180	10	97.70
18	−0.5	0.289	0.204	0.791	2	50	150	30	62.00
19	0	−0.577	0.204	0.791	2.5	35	150	30	50.20
20	0	0	−0.613	0.791	2.5	45	60	30	44.00
21	0	0	0	0	2.5	45	120	20	71.06
22	0	0	0	0	2.5	45	120	20	71.68
23	0	0	0	0	2.5	45	120	20	70.85

The concentration of the residual lead(II) in solutions was determined by atomic absorption spectrophotometry (AAS; PE AAnalyst 800) and the adsorption rate (*Y*) was calculated as the equation (1):

1 $$Y=\,\frac{{C}_{i}-{C}_{r}}{{C}_{i}}\times 100 \% $$

where *C*_*i*_ was the initial lead(II) concentration in mg/L and *C*_*r*_ was the residual lead(II) concentration determined by AAS in mg/L.

Data acquired from Doehlert designs were analyzed by Design-Expert 10 (Stat-Ease, Inc., U.S.A.) and 0.05 was applied as the significant level.

#### Kinetic studies

A good correlation of the kinetic data explained the adsorption mechanism of the metal ion on the solid phase^36^. To evaluate the kinetic mechanism, kinetic studies were conducted in the temperature of 45 °C with the pH of 3. The initial lead(II) concentration was 20 mg/L and adsorption time was the variable, from 10 min, 20 min, 30 min, 60 min to 120 min, 180 min, 240 min and 300 min. The pseudo-first-order and pseudo-second-order models were applied for the kinetic studies.

The pseudo-first-order^37^ model was shown in equation (2):

2 $$\mathrm{log}({q}_{e}-{q}_{t})=\,\mathrm{log}\,{q}_{e}-\frac{{k}_{1}}{2.303}t$$

where *q*_e_ and *q*_t_ (mg/g) were the amount of adsorbed lead(II) at equilibrium and at time *t*; *k*_1_ (min^−1^) was the rate constant of the pseudo-first-order model. Through drawing the plot of $\mathrm{log}({q}_{e}-{q}_{t})$ vs. *t*, *q*_e_ and *k*_1_ were calculated as the slope and intercept and it was required that the calculated equilibrium adsorption capacity value, *q*_e_ (cal.), should be in accordance with the experimental *q*_e_ (exp.) value^38^.

Equation (3) gave the model of the pseudo-second-order^39^:

3 $$\frac{t}{{q}_{t}}=\frac{1}{{k}_{2}{q}_{e}^{2}}+\frac{t}{{q}_{e}}$$

where *k*_2_ (g mg^−1^ min^−1^) was the rate constant of pseudo-second-order adsorption. From the slope and intercept of the plots $\frac{t}{{q}_{t}}$ vs. *t*, the pseudo-second-order rate constant *k*_2_ and *q*_e_ values were acquired.

#### Isotherm studies

The adsorption isotherm provided the relationship between the amounts adsorbed by a unit weight of adsorbent and the amount of adsorbate remaining in aqueous solutions at equilibrium^12^.

In this research, isotherm studies were carried out in lead(II) concentrations from 10 mg/L to 30 mg/L as the initial concentration, and pH, adsorption temperature along with adsorption time were selected accordingly with values of 2.5, 30 °C and 210 min. Langmuir model and Freundlich model were tested to figure out the better one.

Langmuir model^40^ was calculated as the equation (4):

4 $${q}_{e}=\frac{{q}_{max}b{C}_{e}}{1+b{C}_{e}}$$

where *q*_*e*_ (mg/g) was the amount of lead(II) bound to per gram of the pomelo peel at equilibrium, *C*_*e*_ (mg/L) was the residual (equilibrium) lead(II) concentration left in the solution after binding, *q*_*max*_ (mg/g) was the maximum amount of lead(II) per unit weight of the pomelo peel to form a complete monolayer on the surface and *b* (L/mg) was the constant related to the affinity of the binding sites^12^.

The essential characteristics of Langmuir model was explained in terms of dimensionless constant separation factor (*R*_*L*_), defined by equation (5):

5 $${R}_{L}=\frac{1}{1+b{C}_{0}}$$

where *b* (L/mg) was the Langmuir constant and *C*_0_ (mg/L) was the initial concentration of lead(II).

Freundlich model was expressed as equation (6)^41^:

6 $$\mathrm{log}\,{q}_{e}=\,\mathrm{log}\,{K}_{F}+\frac{1}{n}\,\mathrm{log}\,{C}_{e}$$

where *K*_*F*_ and *n* were Freundlich isotherm constants related to biosorption capacity and intensity of adsorption. If the equation (6) worked, a plot of log*q*_e_ versus log*C*_e_ would give a straight line of slope $\frac{1}{n}$ and intercept *K*_F_.

#### Interfering study

10 times concentration of Ca^2+^ and Mg^2+^ with the value of 200 mg/L and the same concentration of Cu^2+^ and Zn^2+^ as Pb^2+^ with the value of 20 mg/L were designed for interfering study to test the selectivity of pomelo peels on absorbing lead(II). Temperature and pH were 45 °C and 3, respectively. The adsorption time was set according to the result of kinetic studies when the solution reached equilibrium.

Interfering study was carried out for three times and the average adsorption rate was chosen to compare and evaluate.

#### SEM analysis

Micrographs of pomelo peels before and after lead(II) adsorption were acquired with a E-1010 Ion Sputter scanning electron microscopy (Hitachi) for the characterization of the adsorbent as well as the elucidation of the probable mechanism of adsorption^12^.

#### FT-IR analysis

Spectra of pomelo peels before and after lead(II) adsorption were recorded with a Nicolet™ iS™ 10 FT-IR spectrophotometer (Thermo Scientific) using KBr pellets methods to discover the functional groups.
